# Origin and insertion of the medial patellofemoral ligament: a systematic review of anatomy

**DOI:** 10.1007/s00167-016-4272-1

**Published:** 2016-09-08

**Authors:** Arash Aframian, Toby O. Smith, T. Duncan Tennent, Justin Peter Cobb, Caroline Blanca Hing

**Affiliations:** 1 Trauma and Orthopaedics Department, 5th Floor St James’ Wing, St George’s University Hospitals NHS Foundation Trust, Blackshaw Road, London, SW17 0QT UK; 2 St George’s, University of London, London, SW17 0RE UK; 3 Imperial College, London, W6 8RP UK; 4 Faculty of Medicine and Health Sciences, University of East Anglia, Norwich, NR4 7TJ UK

**Keywords:** Medial patellofemoral ligament, Anatomy, Insertion, Origin, MPFL, Attachments, Reconstruction

## Abstract

**Purpose:**

The medial patellofemoral ligament (MPFL) is the major medial soft-tissue stabiliser of the patella, originating from the medial femoral condyle and inserting onto the medial patella. The exact position reported in the literature varies. Understanding the true anatomical origin and insertion of the MPFL is critical to successful reconstruction. The purpose of this systematic review was to determine these locations.

**Methods:**

A systematic search of published (AMED, CINAHL, MEDLINE, EMBASE, PubMed and Cochrane Library) and unpublished literature databases was conducted from their inception to the 3 February 2016. All papers investigating the anatomy of the MPFL were eligible. Methodological quality was assessed using a modified CASP tool. A narrative analysis approach was adopted to synthesise the findings.

**Results:**

After screening and review of 2045 papers, a total of 67 studies investigating the relevant anatomy were included. From this, the origin appears to be from an area rather than (as previously reported) a single point on the medial femoral condyle. The weighted average length was 56 mm with an ‘hourglass’ shape, fanning out at both ligament ends.

**Conclusion:**

The MPFL is an hourglass-shaped structure running from a triangular space between the adductor tubercle, medial femoral epicondyle and gastrocnemius tubercle and inserts onto the superomedial aspect of the patella. Awareness of anatomy is critical for assessment, anatomical repair and successful surgical patellar stabilisation.

**Level of evidence:**

Systematic review of anatomical dissections and imaging studies, Level IV.

## Introduction

Patellar dislocation is multi-factorial in aetiology and may involve abnormalities of the bone or soft tissues [82]. The medial patellofemoral ligament (MPFL) is a band of retinacular tissue within layer II of the medial side of knee and as the main medial soft tissue stabiliser of the patella (particularly in early flexion) is critical for tracking and stability in the trochlear groove [38, 66, 69, 81, 88, 101]. Acute lateral patellar dislocation (LPD) is associated with MPFL rupture in 87–100 %, and the significant number of different procedures to treat the problem highlights the limitations in understanding and management [5, 20, 54, 77].

Longer term, patellar instability and dislocation are associated with chondral injury and osteoarthritis [28, 54]. Non-operative management can leave 33 % with significant patient-reported symptoms, 44 % with at least one episode of further dislocation and 52 % of patients unable to return to vigorous sports at an average of 11.8-year follow-up [20]. Surgery can correct recurrent dislocation which untreated can occur in 42–49 % of patients [20, 55].

Reconstruction of the MPFL is now an accepted technique for the treatment of patellofemoral instability when soft tissues rather than bony morphology are the primary pathological feature [18, 29, 74, 88]. Performed non-anatomically, it can lead to non-physiological patellofemoral loads and kinematics which may lead to pain and increased chondral injury [7, 30, 79, 95].

Although Weber and Weber first described the anatomy of the knee in 1836, there remains no consensus on the anatomy of the MPFL or even its existence, which has been reported to be present in 35–100 % of cadaveric specimens [21, 63, 71, 76, 99]. Understanding of MPFL origin and insertion points is fundamental for functional reconstruction of the ligament thus reducing the risk of subsequent repeated instability, maltracking and osteoarthritis [45].

The aim of this study was to conduct a systematic review of the literature to define the anatomy of the MPFL. An accurate definition of MPFL anatomy enables assessment of MPL injuries and planning anatomical reconstruction of the MPFL to restore tracking without overloading the patellofemoral joint.

## Materials and methods

### Search strategy

An electronic PRISMA compliant [59] search was conducted on the 3 February 2016 from database inceptions to the search date. Databases searched included Embase, AMED, Medline, PsycINFO, Cochrane, CINAHL, PubMed and NHS Evidence. Where available, medical subject headings (MeSH) terms were used. In addition, searches of grey literature were conducted using Google Scholar, Web of Science, OpenGrey, Ethos and the Zetoc engines. Search terms used for the MEDLINE search are presented in Table 1. This strategy was modified for the other database searches. The reference lists of all potentially eligible papers were also reviewed to identify any additional studies.

**Table 1:** Search strategy terms for the literature search of anatomy of the medial patellofemoral ligament using MEDLINE (via OVID)

1. Anatomy, Regional/or exp Anatomy/or Anatomy, Cross-Sectional/or Anatomy, Comparative/
2. Exp Knee Joint/or exp Patellofemoral Joint/
3. 1 or 2
4. Medial patellofemoral ligament.mp. [mp = title, abstract, original title, name of substance word, subject heading word, keyword heading word, protocol supplementary concept word, rare disease supplementary concept word, unique identifier]
5. 3 and 4
6. mpfl.mp. [mp = title, abstract, original title, name of substance word, subject heading word, keyword heading word, protocol supplementary concept word, rare disease supplementary concept word, unique identifier]
7. 3 and 6
8. 5 or 7

### Eligibility criteria and identification

**Fig. 1:**
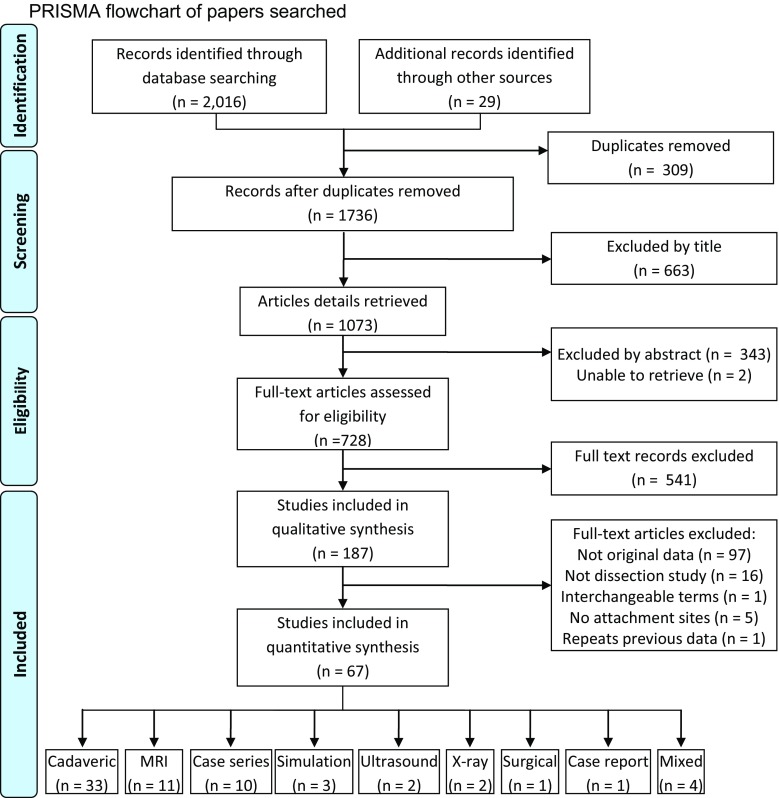
PRISMA flowchart of papers searched

Inclusion eligibility was confirmed if publications investigated the anatomy of the MPFL, specifically origin or insertion points in human subjects. Where inclusion could not be determined from the title and subsequently abstract, the full paper was retrieved as shown in Fig. 1. Assessment of anatomy was permitted either surgically or radiologically. Papers were excluded if MPFL anatomy had been based on other data sources. Studies were eligible irrespective of language, age or country of origin.

The electronic searches were independently performed by two authors (AA, TOS). Based on the eligible criteria, the two reviewers (AA, TOS) independently screened the search results to identify potentially eligible papers. The full-texts of all provisional papers were obtained, and a decision of final eligibility was made after reviewing these by the two reviewers (AA, TOS).

### Data extraction and critical appraisal

Data extraction was made by one reviewer (AA) and repeated by a second reviewer (TOS) to check and verify the findings and accuracy of the results—there was 100 % agreement in data extracted (Fig. 1). All data were extracted into an electronic database. Data extracted included: country of origin, study type, whether the study was of normal or patellar dislocation knees and in cases following dislocation whether acute or recurrent, number of patients and knees, age, gender, preservation method (if cadaveric), MPFL identification rate and size, origin and insertion.

The quality of each included paper was evaluated using a modified critical appraisal skills programme (CASP) tool [89]. This was undertaken by one reviewer (AA) and verified by a second (TOS). Any disagreements in data extraction or quality assessment were resolved through discussion between the reviewers so that there was 100 % agreement in the assessments of cadaveric and radiological or clinical papers (Tables 2, 3).

**Table 2:** Methodological assessment of cadaveric papers

References	Focussed question	Appropriate design	Population defined	[Ethical approval consideration?]	Sample size defined by power	[Source of samples stated]	Outcome measures defined	Dissection method defined	Radiological assessment described	Observers defined	Observer reliability assessed	Multiple observations	Blinding of observer to pathology	Statistical methods described	Variance described	Inferential statistics employed	Appropriate interpretation	Generalisability	Relevance to present evidence base	Clinical relevance discussed	Total number of features met
Philippot et al. [68]	✓	✓	✗	✗	✗	✗	✓	✗	✗	✗	✗	✗	✓	✗	✓	✓	✓	✓	✓	✓	10
Panagiotopoulos et al. [65]	✓	✓	✗	✗	✗	✗	✓	✓	✗	✗	✗	✗	✓	✗	✗	✓	✓	✓	✓	✓	10
Lee et al. [49]	✓	✓	✗	✗	✗	✗	✓	✓	✗	✗	✗	✗	✓	✗	✗	✓	✓	✓	✓	✓	10
Warren and Marshall [101]	✓	✓	✗	✗	✗	✗	✓	✓	✗	✗	✗	✗	✓	✗	✗	✓	✓	✓	✓	✓	10
Reider et al. [76]	✓	✓	✓	✗	✗	✗	✓	✓	✗	✗	✗	✗	✓	✗	✗	✓	✓	✓	✓	✓	11
Tuxøe et al. [98]	✓	✓	✓	✗	✗	✗	✓	✓	✗	✗	✗	✗	✓	✗	✗	✓	✓	✓	✓	✓	11
Feller et al. [32]	✓	✓	✓	✗	✗	✗	✓	✓	✗	✗	✗	✗	✓	✗	✗	✓	✓	✓	✓	✓	11
Starok et al. [91]	✓	✓	✓	✗	✗	✗	✓	✗	✓	✗	✗	✗	✓	✗	✗	✓	✓	✓	✓	✓	11
Schöttle et al. [81]	✓	✓	✗	✗	✗	✗	✓	✗	✓	✗	✗	✗	✓	✗	✓	✓	✓	✓	✓	✓	11
Nomura et al. [62]	✓	✓	✓	✗	✗	✗	✓	✓	✗	✗	✗	✗	✓	✗	✓	✓	✓	✓	✓	✓	12
Triantafillopoulos et al. [97]	✓	✓	✓	✗	✗	✓	✓	✓	✗	✗	✗	✗	✓	✗	✗	✓	✓	✓	✓	✓	12
Andrikoula et al. [3]	✓	✓	✓	✗	✗	✗	✓	✓	✗	✗	✗	✗	✓	✓	✓	✓	✓	✓	✓	✓	13
Mochizuki et al. [57]	✓	✓	✓	✓	✗	✓	✓	✓	✗	✗	✗	✗	✓	✗	✗	✓	✓	✓	✓	✓	13
Viste et al. [99]	✓	✓	✓	✓	✗	✓	✓	✓	✗	✗	✗	✗	✓	✗	✗	✓	✓	✓	✓	✓	13
Nomura et al. [60]	✓	✓	✓	✗	✗	✗	✓	✓	✗	✗	✗	✗	✓	✓	✓	✓	✓	✓	✓	✓	13
Shea et al. [83]	✓	✓	✓	✓	✗	✓	✓	✗	✓	✗	✗	✗	✓	✗	✗	✓	✓	✓	✓	✓	13
Waligora et al. [100]	✓	✓	✓	✓	✗	✓	✓	✓	✗	✗	✗	✗	✓	✗	✗	✓	✓	✓	✓	✓	13
Conlan et al. [21]	✓	✓	✓	✗	✗	✗	✓	✓	✗	✗	✗	✗	✓	✓	✓	✓	✓	✓	✓	✓	13
Jacobi et al. [44]	✓	✓	✓	✗	✗	✗	✓	✓	✗	✗	✓	✓	✓	✗	✓	✓	✓	✓	✓	✓	14
Philippot et al. [69]	✓	✓	✓	✗	✗	✗	✓	✓	✗	✓	✗	✗	✓	✓	✓	✓	✓	✓	✓	✓	14
Smirk and Morris [88]	✓	✓	✓	✗	✗	✓	✓	✓	✗	✗	✓	✗	✗	✓	✓	✓	✓	✓	✓	✓	14
Baldwin. [11]	✓	✓	✓	✓	✗	✗	✓	✓	✗	✗	✗	✗	✓	✓	✓	✓	✓	✓	✓	✓	14
Desio et al. [23]	✓	✓	✓	✗	✗	✗	✓	✓	✗	✗	✓	✓	✗	✓	✓	✓	✓	✓	✓	✓	14
Fujino et al. [37]	✓	✓	✓	✗	✗	✓	✓	✓	✓	✗	✗	✗	✓	✗	✓	✓	✓	✓	✓	✓	14
Aragão et al. [4]	✓	✓	✓	✓	✗	✓	✓	✓	✗	✗	✗	✗	✓	✓	✓	✓	✓	✓	✓	✓	15
Placella et al. [73]	✓	✓	✓	✓	✗	✗	✓	✓	✗	✗	✗	✓	✓	✓	✓	✓	✓	✓	✓	✓	15
Kang et al. [45]	✓	✓	✓	✓	✗	✓	✓	✓	✗	✗	✗	✗	✓	✓	✓	✓	✓	✓	✓	✓	15
Steensen et al. [92]	✓	✓	✓	✗	✗	✗	✓	✓	✓	✓	✗	✗	✓	✓	✓	✓	✓	✓	✓	✓	15
Redfern et al. [75]	✓	✓	✓	✗	✗	✗	✓	✓	✓	✗	✓	✗	✓	✓	✓	✓	✓	✓	✓	✓	15
Barnett et al. [13]	✓	✓	✓	✓	✗	✗	✓	✓	✓	✗	✗	✗	✓	✓	✓	✓	✓	✓	✓	✓	15
Shea et al. [84]	✓	✓	✓	✓	✗	✗	✓	✓	✓	✓	✗	✓	✓	✗	✗	✓	✓	✓	✓	✓	15
LaPrade et al. [48]	✓	✓	✓	✗	✗	✗	✓	✓	✓	✓	✓	✗	✓	✓	✗	✓	✓	✓	✓	✓	15
Farrow et al. [31]	✓	✓	✓	✓	✗	✗	✓	✓	✗	✗	✓	✓	✓	✓	✓	✓	✓	✓	✓	✓	16
Stephen et al. [93]	✓	✓	✓	✓	✗	✗	✓	✓	✓	✗	✓	✓	✓	✓	✓	✓	✓	✓	✓	✓	17

✓ = Satisfies criterion, ✗ = Does not meet criterion

Total score represents number of features met by paper, ascending order

**Table 3:** Methodological assessment of radiological and clinical papers

References	Study type	Focussed Qs	Appropriate design	Population defined	Recruitment methods described	Sample size defined by power	Study setting described	Outcome measures defined	Dissection method defined	Radiological assessment described	Observers defined	Observer reliability assessed	Multiple observations	Blinding of observer to pathology	Statistical methods described	Variance described	Inferential statistics employed	Appropriate interpretation	Generalisability	Relevance to present evidence base	Clinical relevance discussed	Total number of features met
Camanho et al. [17]	Surgical	✓	✓	✗	✗	✗	✗	✗	✓	✗	✗	✗	✗	✗	✗	✗	✗	✓	✓	✓	✓	7
Lippacher and Nelitz [51]	X-ray	✓	✓	✗	✗	✗	✗	✓	✓	✓	✗	✗	✗	✗	✗	✗	✗	✓	✗	✓	✓	8
Matthews and Schranz [55]	Case series	✓	✓	✗	✓	✗	✓	✓	✓	✗	✗	✗	✗	✗	✗	✗	✓	✓	✓	✓	✓	11
Schottle et al. [81]	Mixed	✓	✓	✗	✗	✗	✓	✓	✓	✓	✗	✗	✗	✗	✗	✗	✓	✓	✓	✓	✓	11
Inoue et al. [41]	Case reports	✓	✓	✓	✓	✗	✓	✓	✓	✗	✗	✗	✗	✗	✗	✗	✓	✓	✓	✓	✓	12
Avikainen et al. [8]	Case series	✓	✓	✓	✓	✗	✓	✓	✓	✗	✗	✗	✗	✗	✗	✗	✓	✓	✓	✓	✓	12
Felus et al. [34]	USS	✓	✓	✓	✓	✗	✓	✓	✗	✓	✗	✗	✗	✗	✗	✗	✓	✓	✓	✓	✓	12
Ahmad et al. [1]	Case series	✓	✓	✓	✓	✗	✓	✓	✓	✓	✗	✗	✗	✗	✗	✗	✓	✓	✓	✓	✓	13
Deie et al. [22]	Case series	✓	✓	✓	✓	✗	✓	✓	✓	✓	✗	✗	✗	✗	✗	✗	✓	✓	✓	✓	✓	13
Nomura et al. [59]	Mixed	✓	✓	✓	✓	✗	✓	✓	✓	✓	✗	✗	✗	✗	✗	✗	✓	✓	✓	✓	✓	13
Dirim et al. [25]	Mixed	✓	✓	✓	✗	✗	✓	✓	✓	✓	✓	✗	✗	✗	✗	✗	✓	✓	✓	✓	✓	13
Phornphutkul et al. [70]	Mixed	✓	✓	✓	✗	✗	✓	✓	✓	✓	✓	✗	✗	✗	✗	✗	✓	✓	✓	✓	✓	13
Sallay et al. [77]	MRI	✓	✓	✓	✓	✗	✓	✓	✓	✓	✗	✗	✗	✗	✗	✗	✓	✓	✓	✓	✓	13
Kang et al. [46]	MRI	✓	✓	✗	✓	✗	✓	✓	✗	✗	✗	✓	✗	✗	✓	✓	✓	✓	✓	✓	✓	13
Balcarek et al. [9]	MRI	✓	✓	✓	✓	✗	✓	✓	✗	✓	✗	✗	✗	✗	✓	✗	✓	✓	✓	✓	✓	13
Iwama et al. [42]	MRI	✓	✓	✓	✓	✗	✓	✓	✗	✓	✓	✗	✗	✗	✗	✗	✓	✓	✓	✓	✓	13
Nomura and Inoue [61]	Case series	✓	✓	✓	✓	✗	✓	✓	✓	✓	✗	✗	✗	✗	✗	✓	✓	✓	✓	✓	✓	14
Balcarek et al. [10]	MRI	✓	✓	✓	✓	✗	✓	✓	✗	✓	✗	✗	✗	✗	✓	✓	✓	✓	✓	✓	✓	14
Wissman et al. [103]	MRI	✓	✓	✓	✓	✗	✓	✓	✗	✓	✓	✗	✓	✗	✗	✗	✓	✓	✓	✓	✓	14
Oka et al. [63]	Simulation	✓	✓	✓	✓	✗	✓	✓	✗	✓	✗	✗	✗	✗	✓	✓	✓	✓	✓	✓	✓	14
Christiansen et al. [19]	Case series	✓	✓	✓	✓	✓	✓	✓	✓	✗	✗	✗	✗	✗	✓	✓	✓	✓	✓	✓	✓	15
Lim et al. [50]	Case series	✓	✓	✓	✓	✗	✓	✓	✓	✓	✗	✗	✗	✗	✓	✓	✓	✓	✓	✓	✓	15
Enderlein et al. [30]	Case series	✓	✓	✓	✓	✗	✓	✓	✓	✓	✗	✗	✗	✗	✓	✓	✓	✓	✓	✓	✓	15
Elias et al. [28]	MRI	✓	✓	✓	✓	✗	✓	✓	✗	✓	✓	✗	✓	✗	✓	✗	✓	✓	✓	✓	✓	15
Graf et al. [39]	Simulation	✓	✓	✓	✓	✗	✓	✓	✗	✓	✗	✗	✓	✗	✓	✓	✓	✓	✓	✓	✓	15
Bitar et al. [15]	Case series	✓	✓	✓	✓	✓	✓	✓	✓	✓	✗	✗	✗	✗	✓	✓	✓	✓	✓	✓	✓	16
Kepler et al. [47]	MRI	✓	✓	✓	✓	✗	✓	✓	✗	✓	✗	✓	✓	✗	✓	✓	✓	✓	✓	✓	✓	16
De Oliveira et al. [64]	MRI	✓	✓	✓	✓	✗	✓	✓	✗	✓	✓	✓	✗	✗	✓	✓	✓	✓	✓	✓	✓	16
Yoo et al. [104]	Simulation	✓	✓	✓	✓	✗	✓	✓	✗	✓	✓	✗	✓	✗	✓	✓	✓	✓	✓	✓	✓	16
Zhang et al. [105]	USS	✓	✓	✓	✓	✗	✓	✓	✗	✓	✓	✓	✗	✗	✓	✓	✓	✓	✓	✓	✓	16
Tateishi et al. [94]	Case series	✓	✓	✓	✓	✗	✓	✓	✓	✓	✗	✓	✓	✗	✓	✓	✓	✓	✓	✓	✓	17
Sillanpää et al. [86]	MRI	✓	✓	✓	✓	✗	✓	✓	✗	✓	✓	✗	✓	✓	✓	✓	✓	✓	✓	✓	✓	17
Sillanpää et al. [87]	MRI	✓	✓	✓	✓	✗	✓	✓	✓	✓	✗	✓	✓	✓	✓	✓	✓	✓	✓	✓	✓	18
Wijdicks et al. [102]	X-ray	✓	✓	✓	✓	✗	✓	✓	✓	✓	✓	✓	✓	✗	✓	✓	✓	✓	✓	✓	✓	18

✓ = Satisfies criterion, ✗ = Does not meet criterion

Total score represents number of features met by paper, ascending order

### Data analysis

Due to multiple methodologies used in the included studies, there was a high degree of study heterogeneity, and therefore, a meta-analysis was inappropriate. Accordingly, a narrative analysis was adopted to determine the consensus on MPFL identification rate, MPFL size, and origin and insertion of the MPFL from the study cohorts.

## Results

### Search results

A summary of the search results is presented in Fig. 1. A total of 2045 papers were identified. Of the 187 potentially relevant papers, 67 satisfied the inclusion criteria.

### Characteristics of included studies

The 67 studies included 1950 knees where the MPFL was investigated in over 1475 patients (some cadaveric studies did not state whether the knees were from the same body). Where both the average age and number of patients were given (35 studies), the weighted average age was 33 years. Gender was given in 44 publications with 613 males (48 %) and 658 females (52 %). The 67 studies were from several countries and included 764 (39 %) normal, 507 (26 %) acute dislocation, 381 (20 %) recurrent dislocation and 289 (15 %) mixed pathology knees. Investigation technique was heterogeneous (Tables 4, 5).

**Table 4:** Patellar insertions of the MPFL reported

Site	References	Number of knees
Medial patella	Panagiotopoulos et al. [65]^a^	8
	Mochizuki et al. [57]^a^	16
	Lee et al. [49]^a^	5
	Starok et al. [91]^a^	5
	Waligora et al. [100]^a^	18
	Matthews and Schanz [55]^e^	21
	Iwama et al. [42]^c^	25
	Kang et al. [46]^c^	85
	Sillanpää et al. [86]^c^	32
	Sillanpää et al. [87]^c^	56
Proximal	Viste et al. [99]^a^	12
	Wissman et al. [103]^c^	10
Proximal half	Jacobi et al. [44]^a^	20
	Philipott et al. [69]^a^	23
	Conlan et al. [21]^a^	25
	Stephen et al. [93]^a^	8
Proximal one third	Triantafillopoulos et al. [97]^a^	8
	Placella et al. [73]^a^	20
	Tateishi et al. [94]^e^	27
	Graf et al. [39]^d^	10
Proximal one third to midline	Nomura et al. [60]^a^	17
	Nomura et al. [59]^b^	27
Middle	Kang et al. [45]^a^	12
	Shea et al. [84]^a^	9
	Yoo et al. [104]^d^	10
Proximal two thirds	Tuxøe et al. [98]^a^	39
	Andrikoula et al. [3]^a^	10
	Nomura et al. [62]^a^	20
	Baldwin. [11]^a^	50
	Inoue et al. [41]^f^	2
	Christiansen et al. [19]^e^	42
	Balcarek et al. [10]^c^	73
	Balcarek et al. [9]^c^	43
Superomedial	LaPrade et al. [48]^a^	8
	Feller et al. [32]^a^	20
	Desio et al. [23]^a^	9
	Barnett et al. [13]^a^	10
	Hautamaa et al. [40]^a^	17
	Phornphutkul et al. [70]^b^	5
	Dirim et al. [25]^b^	12
	De Oliveira et al. [64]^c^	125
	Zhang et al. [105]^g^	49
	Steensen et al. [92]^a^	11
Insertion fans out over patella and surrounding tissues	Fujino et al. [37]^a^	31
	Smirk and Morris [88]^a^	25
	Aragão et al. [4]^a^	10

Study type: ^a^Cadaveric; ^b^Mixed; ^c^MRI; ^d^Simulation; ^e^Series; ^f^Case report; ^g^USS

**Table 5:** Femoral origins of the MPFL reported

Site	Papers	Number of knees
Adductor tubercle (AT)	Starok et al. [91]^a^	5
Distal to AT	Smirk and Morris [88]^a^	25
	Viste et al. [99]^a^	12
	Jacobi et al. [44]^a^	20
	Nomura et al. [60]^a^	17
	Tuxøe et al. [98]^a^	39
	Nomura et al. [62]^a^	20
	Dirim et al. [25]^b^	12
	Lim et al. [50]^b^	27
Medial femoral epicondyle (MFE)	Panagiotopoulos et al. [65]^a^	8
	Mochizuki et al. [57]^a^	16
	Andrikoula et al. [3]^a^	10
	Steensen et al. [92]^a^	11
	Reider et al. [76]^a^	48
	Hautamaa et al. [40]^a^	17
	Kang et al. [46]^c^	85
Between MFE and AT	Philippot et al. [69]^a^	23
	Placella et al. [73]^a^	20
	Laprade et al. [48]^a^	8
	Fujino et al. [37]^a^	31
	Stephen et al. [93]^a^	8
	Baldwin [11]^a^	50
	Farrow et al. [31]^a^	16
	Lee et al. [49]^a^	5
	Waligora et al. [100]^a^	18
	Kang et al. [45]^a^	12
	Barnett et al. [13] ^a^	10
	Schöttle et al. [81]^a^	8
	De Oliveira et al. [23]^c^	125
	Sillanpää et al. [86]^c^	32
	Balcarek et al. [10]^c^	73
	Balcarek et al. [9]^c^	43
	Yoo et al. [104]^d^	10
	Enderlein et al. [30]^e^	240
	Tateishi et al. [94]^e^	27
	Bitar et al. [15]^e^	41
	Wijdicks et al. [102]^f^	11
	Nomura et al. [59]^b^	27
MFE and AT	Iwama et al. [42]^c^	25
	Aragão et al. [4]^a^	17
	Triantafillopoulos et al. [97]^a^	8
Anterior to MFE	Feller et al. [32]^a^	20
Adjacent to medial collateral (MCL) insertion	Deie et al. [22]^e^	6
AT with MCL	Phornphutkul et al. [70]^b^	5
Between AT and MCL	Christiansen et al. [19]^e^	42
	Avikainen et al. [8]^e^	14
	Wissman et al. [103]^c^	10
With superficial MCL	Warren and Marshall [101]^a^	154
AT and MFE and superficial MCL	Desio et al. [23]^a^	9
Over MFE, distal to AT, partly to capsule	Inoue et al. [41]^g^	1
38 % of postero-anterior distance and 54 % of distal-proximal distance of condyle	Oka et al. [63]^d^	20
5 mm anterior to posterior cortex, 3 mm proximal to apex with Blumensaat’s line	Redfern et al. [75]^a^	8
Proximal to physis	Shea et al. [83]^a^	6
5-6 mm distal to physis	Kepler et al. [47]^c^	44
	Lippacher and Nelitz [51]^f^	27

Study type: ^a^Cadaveric; ^b^Mixed; ^c^MRI; ^d^Simulation; ^e^Series; ^f^X-ray; ^g^Case report

*AT* adductor tubercle, *MFE* medial femoral condyle, *MCL* medial collateral ligament

### Characteristics by study design

#### Surgical studies

Surgical technique or case series reports usually describe an approach rather than study anatomy (Tables 4, 5). Five studies were of acute patellar dislocation, and seven referred to recurrent patellar dislocation. Amongst the ten papers which did specify gender, nine papers included 76 male and 77 female subjects and a single case series which reported 78 male and 162 female knees, but only 224 participants indicating that some must be from the same participants [32]. Four studies were in adults, six were mixed age groups and one did not give the age of subjects [17]. One study looked at MPFL reconstruction in children [24]. Eight papers provided the average age, giving a weighted average of 21 years for adults (9 years for children). Due to expectation bias of surgical studies, these were excluded from the final analysis.

#### Imaging studies

Amongst the imaging studies, there was also heterogeneity (Tables 4, 5). Eight studies were of anatomy in acute patellar dislocation, one with recurrent patellar dislocation, one in patients without any history or evidence of patellofemoral instability and five studies were a mixture of these types. There was a large variation in subjects, with an age range of 12–89 years, and average ages of 14–72 years even within imaging modalities with the two radiographic studies reporting a large variation in age range [53, 102]. Another study which correlated magnetic resonance imaging (MRI) findings to histology reported that in two of five knees examined, the MPFL attached to the medial femoral epicondyle (MFE) with the medial collateral ligament (MCL) [27].

#### Cadaveric studies

The methodological assessment of the 33 cadaveric studies is shown in Table 2. Cadaveric studies were in knees which the authors believed to be normal, and different cadaveric preservation techniques were used, with eighteen fresh frozen, eight embalmed, two unpreserved and the remainder using more than one preservation method (five papers) or not describing the preservation technique used (four papers).

To determine possible variations in anatomy (particularly knee size and MPFL length) based on height of subjects or cadavers, the origin countries for papers were recorded, and particularly if they described the source of the cadavers for dissection studies. Only nine of the 33 cadaveric studies specifically stated where the knees were sourced from. For the others, even if all authors were from the same country, then this was recorded separately as cadavers may be obtained and transported from a different country.

In total amongst the 33 studies, there were 705 cadaveric knees (range 5–154, median 16). These were mostly adult knees (29/33 papers) given that paediatric cadavers (3/33 papers) are not readily available [33]. One study did not specify the age range [51]. For the purposes of this review, skeletal maturity was defined as closure of the distal femoral physis. Amongst the adult cadaveric studies, the median of the average ages reported amongst the 18 papers that provided this information was 71 (range 19–100) years and when number of patients was also given (12 papers), the weighted average was 75 years in this subset. Cadaveric specimens do not accurately reflect the age of the patients typically seen with patellar dislocation or instability as demonstrated by the largest case series in this review of 224 patients with a median age of 23 years [32]. Likewise, of the three paediatric groups, one was of children aged 1 month–11 years [83].

There were also discrepancies with gender representation in cadaveric studies. Of the studies of adults, only 18/33 recorded the gender of subjects (158 male, 121 female), in contrast to the usual finding in patellofemoral instability of more female than male patients [37]. There was no significant difference in laterality of knees in the cadaveric papers.

### Quality assessment

The results of the CASP assessment are presented in Tables 2, 3. The overall quality of the evidence was strong with several studies scoring highly in the quality analysis and overlap of results.

Recurrent weaknesses of the cadaveric literature were that none of the studies were based on a sample size calculation, the source of the cadaveric samples were only presented in 26 % of papers and the observer or assessor was only defined in 12 % of papers. Only two studies amongst the radiological and clinical based their sample size on a power calculation, the assessor was only defined in 29 % of papers, observer reliability was ascertained in 21 % of papers, and only 36 % of studies recorded measures from multiple observations (Table 3).

### Anatomy of MPFL synthesis findings

#### MPFL identification rate

Data on MPFL identification rate were given in 28 studies. Earlier studies reported that the MPFL was not identified in all knees, raising a question about whether it was always present. In this systematic review, documented identification of the MPFL varied from 35 to 92 % in four studies, 24 studies described 100 % identification of the MPFL and five did not specifically state the rate of identification, but did not report difficulty identifying the ligament.

#### MPFL size

Data on MPFL length were presented in 19 papers. There was no obvious relationship to country of origin, or method of study with both the shortest (45 mm) and the longest (74 mm) reported MPFL lengths being from Japan and China, respectively. A single USS review article had reported a length of 40 mm but did not state whether this value was actually measured and was therefore excluded [22]. The weighted MPFL mean length and the unweighted median of the averages were both 56 mm. The reliability of reported length is in part dependent on the difficulty in measuring it. More specifically, it has a broad insertion onto the patella 20–30 mm wide, being largely on the proximal medial border, with some fibres extending to the lower third in a minority of cases [65]. Similarly, the femoral origin of the ligament covers an area of around 10–22 mm width [65]. With such broad attachment points, it is difficult to know whether the different studies were measuring the same length.

An MRI study reported a 0.1 mm greater thickness of the MPFL in men than in women (1.0 and 0.9 mm, respectively), whilst another reported statistically significant (*p* < 0.001) increasing MPFL length when comparing normal, unstable and recurrently dislocating patellae at 49, 54 and 64 mm, respectively [23, 44]. In contrast, MPFL lengths from 47 to 72 mm were reported in normal knees so it is unclear whether there is a direct correlation between patellar stability and ligament length, particularly given differences in measurement methods [65, 72]. This difference was also not explained by source of specimens (both cadaveric) or preservation method (both fresh frozen).

Investigating normal and acute MPFL injury MRI scans, a Japanese study reported average lengths of 53 and 49 mm in normal men and women, whereas a Chinese publication reported average MPFL length of 59 mm in acute patellar dislocation [44, 48]. This would seem, then, to support the concept that MPFL length is increased in patellar dislocation. What is not known is whether these patients dislocate because they had longer MPFLs or whether they had longer MPFLs because they have dislocated and the injured MPFL heals longer than it was prior to injury. There was evidence that the MPFL ruptured once elongated beyond 12–18 mm and so even a first episode of patellar dislocation would result in injury [2]. Although the Chinese paper did not provide length separately by gender, the distribution of participants in papers was not dissimilar, being 4:6 and 32:53 males to females, respectively.

#### Origin and insertion of the MPFL from the study cohorts

There were only four paediatric studies and they were not easily comparable as one related to the patellar attachment alone and another publication by the same authors was related to the physis alone [83, 84]. Of the two remaining paediatric papers, one reported attachment next to the superficial MCL attachment and the other in the sulcus between the adductor tubercle (AT) and the medial femoral epicondyle (MFE), which covers a similar area [24, 33].

Fifteen of the studies were of acute patellar dislocation, forty in normal knees, nine in recurrent dislocation and three were a mixture of acute and recurrent. Again, there was no significant difference in attachment points reported, with a mixture of locations given in each group.

**Fig. 2:**
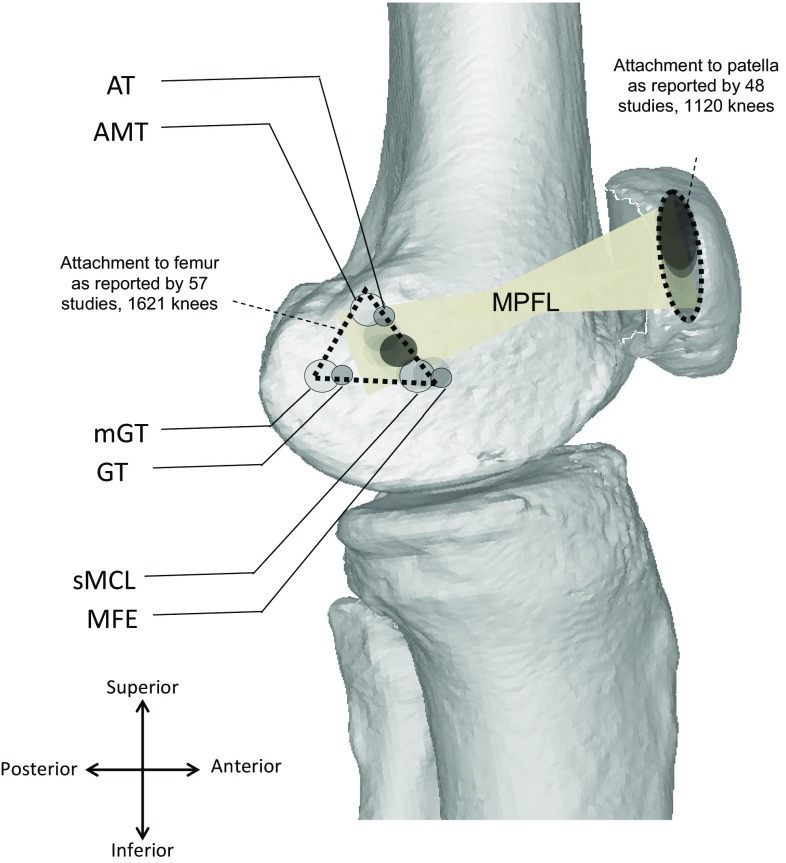
Diagram summarising the MPFL attachment areas, darker shading represents study concordance. *AT* adductor tubercle, *AMT* adductor magnus tendon, *GT* gastrocnemius tubercle, *mGT* medial gastrocnemius tendon, *sMCL* superficial medial collateral ligament, *MFE* medial femoral condyle

Data on MPFL femoral origin were given in 33 papers and MPFL patellar insertion in 29 papers (Tables 4, 5). There was excellent correlation on the insertion of the MPFL onto the medial patella but some discordance regarding which part precisely (Table 4; Fig. 2). There is evidence that the MPFL fans out and may have attachments distributed along the medial border, with 13 % upper third only, seven percent middle third only, 40 % upper and middle thirds, 13 % middle and lower thirds and 27 % along the whole length [4]. The patellar insertion measures 24 ± 5 (standard deviation, SD) mm, and given that the articular surface of the patella is 46 mm long, there was overlap in reports of the patellar insertion (the insertion is half the length of the articular surface) [12, 67].

The greatest variations in anatomy described were of the femoral origin of the MPFL, with several sites reported in the literature (Table 5; Fig. 2). The size of the femoral origin itself varied with the width reported as 9–17 mm [4, 65, 72]. Compared to the width of the MFE and length of the MPFL itself, this is relatively large with the femoral attachment covering an area rather than a discrete point. To compare normal cadaveric knees to those with recurrent dislocation, the literature relevant to surgery for recurrent dislocation was reviewed. Many were excluded through screening as they referred to the MPFL origin without stating where this was found, or citing other papers [40, 85]. Where given, the attachment was within similar boundaries and covered an area that was more posterior and proximal to the medial epicondyle [17].

## Discussion

The most important finding of this review was that the MPFL originates between the medial femoral epicondyle, adductor tubercle and gastrocnemius tubercle and inserts on to the superomedial aspect of the patella with an average length of 56 mm.

Studies have shown that a non-anatomical surgical reconstruction can lead to aberrant restraining forces and patellofemoral contact pressures [14, 26, 30]. Mal-positioning of the femoral insertion in the distal or proximal plane has been shown to have the most significant effect on isometry [93]. However, authors investigating the isometric and non-isometric attachment sites at the medial femoral epicondyle and adductor tubercle, respectively, did not find a significant difference in the contact pressures [57] and this may be explained by the relatively large attachment points that we have found with our systematic review. Use of the attachment areas (Fig. 2) rather than a specific point may be a truer representation of the normal anatomy, and this may provide a safe working window for graft attachment during MPFL reconstruction.

The literature search revealed no prior systematic reviews specifically investigating MPFL anatomy, and we are aware of only one review of the anatomy in 2015 with several more studies published on the subject in the interim. Their review had a number of limitations. The papers reviewed were limited chronologically to the 20 years prior to the literature search with a more limited search of the grey literature performed. Although the authors describe the mean length and the approximate area of the femoral attachment, there was no consensus given and it was difficult to translate this conclusion into a meaningful surgical planning tool [73]. Further, they stated that the MPFL had a ‘sail type’ shape, but in our wider review of the literature above, we have shown that it not only fans out at the patellar insertion but also at the femoral insertion, with a narrow central portion and is therefore shaped like an hourglass (Fig. 2).

Some issues also arise from nomenclature as the terminal part of the medial ridge of the linea aspera of the femur becomes the adductor tubercle. Previous studies have reported that publications have described the attachments interchangeably [11, 65] (regarding [93, 98]) [80].

Many of the studies looked at normal knees, not knees with patellar instability, and this may not be a true reflection of knees where the MPFL has been injured. Previous epidemiological studies demonstrated that patellar dislocation typically occurs during adolescence yet all but a handful of specifically paediatric studies using cadavers were in elderly adults [37, 96]. There may be age-related changes in the ligament which should be considered given that some cases of recurrent instability are reported to improve without surgical intervention [6]. Within paediatric studies, there were differences in reporting (if measured proximal or distal to the physis) due to concavity of the growth plate [53]. Cadaveric studies do not reflect the gender bias with a 3:1 risk ratio for females to males aged 10–17 years for previous subluxation or dislocation, whereas cadaveric specimens were generally gender-balanced [37].

Imaging studies also have limitations, with both CT and MRI affected by partial volume loss between axial slices when the raw image data are obtained, and further losses within each slice from averaging algorithms. In addition, there was bias with more positive identification in patients with patellar dislocation or injury because the MPFL is more easily identified in patients with an effusion than in control patients [31]. In specialist studies, identification or exclusion of MPFL injuries using USS has been shown to have 100 % sensitivity, specificity, negative and positive predictive values compared to subsequent surgical findings, and has the added advantage of dynamic imaging to look at the integrity of the ligament with respect to function [35].

Whilst studies have shown that the femoral fixation point in MPFL reconstruction is more important than the patellar fixation point, a non-anatomical femoral fixation point is not in isolation predictive of graft failure. Sanchis-Alfonso et al. investigated the influence of femoral fixation site on ligament dynamic changes and clinical outcome in 24 patients with 3D CT reconstruction. They found that out of 24 patients with a non-anatomical femoral graft fixation site, only four were defined as failures. Out of these four failures, three had anterior knee pain and only one had recurrent instability. They concluded that whilst reproducing an anatomical femoral fixation point was a reproducible way of achieving an optimal result, a non-anatomical femoral fixation site that reproduced graft isometry specifically during 0°–30° of flexion will still produce a satisfactory result [78]. This can also be explained by our study that has shown that the MPFL is hourglass in shape which may explain the satisfactory results obtained with previous studies investigating a ‘non-anatomic’ graft placement.

Warren and Marshall reported that the MPFL forms the inferior part of an anatomical space of triangular shape formed by the MPFL with the adductor magnus tendon (AMT) and the vastus medialis obliquus (VMO), with the femoral origin lying between MFE and AT [101], although the MPFL was not always easily identified.

One cadaveric dissection study included inspection for collateral knee structures in 20 cases, finding 13 had a lateral patellofemoral ligament (LPFL), and six of these also had a MPFL, whereas one specimen had a MPFL only [76]. They reported a finding of MPFL breadth of 5–12 mm in seven specimens. The MPFL superior border meets the oblique distal fibres of the vastus medialis obliquus, and there is wide variation in angle and differing origins and insertions, so there may be an underestimation of how often the MPFL is present. A 2008 study reported the ligament present in 15 of 17 cases (88 %) [4]. More recent studies have reported that the MPFL can be very thin, but is consistently seen at dissection [2, 16].

Knowledge of the anatomical origin and insertion points enables accurate assessment of potential injury and surgical repair or reconstruction, particularly in recurrent patellar dislocation. Understanding of key anatomical differences between normal and dislocation groups can guide surgical planning where lack of attention and understanding of attachment sites has been linked to a significant proportion of poor outcome in patients undergoing surgery [90].

Future studies with larger numbers of demographically linked (younger, proportionately more female) patients with and without patellar instability would be useful to confirm the findings of this systematic review. Whilst the instability group may be more easily available for surgical exploration, this will not represent the normal MPFL given the almost universal MPFL rupture rate from a single dislocation episode and the high rates of trochlear dysplasia.

## Conclusion

The MPFL is hourglass in shape, originates from the medial femur and inserts onto the medial patellar border with an average length of 56 mm.

## Abbreviations

AA: Arash Aframian
AMT: Adductor magnus tendon
AT: Adductor tubercle
CASP: Critical appraisal skills programme
CH: Caroline Hing
CT: Computed tomography
DT: Duncan Tennent
MeSH: Medical subject headings
JC: Justin Cobb
LPD: Lateral patellar dislocation
LPFL: Lateral patellofemoral ligament
MCL: Medial collateral ligament
MFE: Medial femoral epicondyle
MPFL: The medial patellofemoral ligament
MRI: Magnetic resonance imaging
PRISMA: Preferred reporting items for systematic reviews and meta-analyses
USS: Ultrasound scan
SD: Standard deviation
TOS: Toby O Smith
VMO: Vastus magnus obliquus
